# Gain-of-Function Mutations R249C and S250C in Complement C2 Protein Increase C3 Deposition in the Presence of C-Reactive Protein

**DOI:** 10.3389/fimmu.2021.724361

**Published:** 2021-11-25

**Authors:** Aleksandra Urban, Daria Kowalska, Grzegorz Stasiłojć, Alicja Kuźniewska, Anna Skrobińska, Emilia Arjona, Eugenia Castellote Alonso, María Ángeles Fenollosa Segarra, Ilse Jongerius, Robbert Spaapen, Simon Satchell, Marcel Thiel, Stanisław Ołdziej, Santiago Rodriguez de Córdoba, Marcin Okrój

**Affiliations:** ^1^ Department of Cell Biology and Immunology, Intercollegiate Faculty of Biotechnology of University of Gdańsk and Medical University of Gdańsk, Gdańsk, Poland; ^2^ Centro de Investigaciones Biológicas and Centro de Investigación Biomédica en Enfermedades Raras, Madrid, Spain; ^3^ Servicio Nefrología, Consorci Hospitaliri de Vic, Barcelona, Spain; ^4^ Servicio de Nefrología, Hospital General Universitario de Castellón, Castellón, Spain; ^5^ Department of Immunopathology, Sanquin Research, Amsterdam and Landsteiner Laboratory, Amsterdam University Medical Center, University of Amsterdam, Amsterdam, Netherlands; ^6^ Emma Children’s Hospital, Department of Pediatric Immunology, Rheumatology and Infectious Diseases, Amsterdam University Medical Center, Amsterdam, Netherlands; ^7^ Bristol Renal, Bristol Medical School, University of Bristol, Bristol, United Kingdom; ^8^ Laboratory of Biopolymers Structure, Intercollegiate Faculty of Biotechnology of University of Gdańsk and Medical University of Gdańsk, Gdańsk, Poland

**Keywords:** complement system, aHUS, C3 glomerulopathy, complement C2, endothelial cells

## Abstract

The impairment of the alternative complement pathway contributes to rare kidney diseases such as atypical hemolytic uremic syndrome (aHUS) and C3 glomerulopathy (C3G). We recently described an aHUS patient carrying an exceptional gain-of-function (GoF) mutation (S250C) in the classical complement pathway component C2 leading to the formation of hyperactive classical convertases. We now report the identification of the same mutation and another C2 GoF mutation R249C in two other patients with a glomerulopathy of uncertain etiology. Both mutations stabilize the classical C3 convertases by a similar mechanism. The presence of R249C and S250C variants in serum increases complement-dependent cytotoxicity (CDC) in antibody-sensitized human cells and elevates deposition of C3 on ELISA plates coated with C-reactive protein (CRP), as well as on the surface of glomerular endothelial cells. Our data justify the inclusion of classical pathway genes in the genetic analysis of patients suspected of complement-driven renal disorders. Also, we point out CRP as a potential antibody-independent trigger capable of driving excessive complement activation in carriers of the GoF mutations in complement C2.

## Introduction

The etiology of rare kidney diseases such as C3 glomerulopathy (C3G) and atypical hemolytic uremic syndrome (aHUS) involves dysregulation of the complement system (1). The common etiologic factor is impairment of proteins engaged in the alternative complement pathway (AP), as this route is constantly active at a low level, and its further propagation depends on endogenous inhibition on self surfaces. Conversely, the classical and lectin complement pathways (CP/LP) need specific stimuli, and therefore loss of pathway regulation may not be sufficient for the occurrence of pathology. Elements of AP but not CP/LP are included in routine genetic diagnostics of glomerulopathies. Previously, our group identified the first-ever gain-of-function (GoF) mutation in the CP/LP convertase component, C2, in an aHUS patient (2). Substitution of serine 250 to cysteine renders CP/LP convertase insensitive to regulation by CD55 complement inhibitor, which significantly increases generation and deposition of C3 on target cells. Heterozygous S250C mutation in C2 was the only complement pathogenic variant found in this patient, who also carried the homozygous risk polymorphism in the promoter region of the *MCP* gene that encodes membrane-bound complement inhibitor CD46 (2, 3). However, the mechanism that triggers the pathogenic scenario in the S250C mutation carrier remains unknown. Herein, we report the identification of the S250C mutation and another C2 GoF mutation, R249C, adjacent to the S250C mutation, in two other patients with chronic renal disease. We show that both GoF C2 variants increase the deposition of C3 in the presence of C-reactive protein (CRP), an acute phase protein that elevates its concentration in plasma up to 1,000 times upon infection and/or inflammation (4). To investigate the potential contribution of R249C and S250C C2 proteins to the pathogenic mechanism, we have used immortalized glomerular endothelial cell cultures.

## Methods

### Cells

The human lymphoma cell lines Raji and Ramos (both obtained from the American Type Culture Collection, ATCC) were cultured in RPMI 1640 medium with l-glutamine (ATCC) supplemented with 10% FBS (ATCC). Cells were cultivated at 37°C and in humidified 5% CO_2_ atmosphere. Raji cells with CD55 knockout were produced by CRISPR/Cas9 technology as described in (5). Immortalized human glomerular endothelial cells (iGEnC) (6) were cultured in EGM2-MV medium (Lonza) at 33°C to activate the temperature-sensitive SV40LT transgene. Before the experiments, cells were transferred to 37°C and kept for 5 days to ensure that the transgene was inactive.

### Expression and Purification of C2 Variants

All C2 variants used in the current study were produced in HEK293 Freestyle cells (ThermoFisher) as described in (2, 7). Similar to WT and S250C variants previously described, cDNA coding R249C sequence was codon-optimized, codons for six histidine residues were added at 3’, and all matrices were synthesized in the framework of GeneArt Synthesis^®^ service by ThermoFisher. The construct was cloned into a pCEP4 vector and transfected using Freestyle Max reagent (ThermoFisher). One microgram of each C2 protein was separated in 12% polyacrylamide gel electrophoresis in reducing conditions and stained with Coomassie Brilliant blue.

### Patients

Information about rare genetic variants in THBD, DGKE, *C1QA*, *C1QB*, *C1QC*, *C1R*, *C1S*, *C2*, *C3*, *C4A*, *C4BPA*, *C4BPB*, *C5*, *C7*, *C8A*, *C8B*, *C8G*, *C9*, *CD46*, *CD55*, *CD59*, *CFB*, *CFD*, *CFH*, *CFHR1*, *CFHR3*, *CFHR4*, *CFHR5*, *CFI*, *CFP*, *CLU*, *CR1*, *CR2*, *FCN1*, *FCN2*, *FCN3*, *ITGAX*, *ITGB2*, *MASP1*, *MASP2*, *MBL2*, *SERPING1*, *VSIG4*, and *VTN* genes among aHUS and C3G patients were retrieved from the Spanish aHUS/C3G Registry. Diagnostics criteria and detailed methodology of next-generation sequencing with the following data analyses were described in detail in (2).

### CDC and Classical Convertase Assays

Complement-dependent cytotoxicity (CDC) assay was performed in Raji cells as described in (8). Briefly, cells were loaded with 1mM calcein-AM (Sigma) for 30 min at 37°C. Then, cells were washed twice with PBS buffer, plated onto V-shape 96-well plate in the amount 1 × 10^5^ cells/well and overlaid with 25 μl of anti-CD20 antibody ofatumumab (50 μg/ml) and 25 μl of C2-depleted (ΔC2) NHS (Complement Technology, Tyler, TX, USA) supplemented with physiological concentrations of C2 WT, R249C, or S250C mutant. In some experiments, normal human serum (NHS) or a mix of NHS and patient sera were used. After 30 min of incubation at 37°C, the fluorescence of calcein released into the supernatant was measured with Synergy H1 microplate reader (BioTek). The cell lysis was estimated as a percentage of full lysis, i.e., the readout obtained for the cells incubated with 30% DMSO.

Ramos cells were used for classical convertase assays, as in (2). Cells were loaded with calcein-AM as in CDC assay, then overlayed with ofatumumab (50 μg/ml) and 10% C3-depleted (ΔC3) or C5-depleted (ΔC5) NHS (Complement Technology) supplemented with a physiological concentration of C2 variants (WT, R249C, and S250C). Convertase formation was stopped by the addition of EDTA-GVB buffer (40 mM EDTA diluted in 5 mM veronal buffer, 0.1% gelatin, 145 mM NaCl) at indicated time points (15 s, 30 s, 1 min, 2.5 min, 5 min, and 10 min for C3 convertase; 1 min, 2.5 min, 5 min, 10 min, and 20 min for C5 convertase). Cells were washed and suspended in 5% guinea pig serum (Harlan Laboratories) in EDTA-GVB and incubated for 30 min at 37°C with shaking. The readout and calculation of the percentage of cell lysis were performed as in the CDC assay. Heat-inactivated normal human serum (ΔNHS) served as a negative control.

### Fluorescent Microscopy and Flow Cytometry Analysis of iGEnCs

iGEnCs were seeded on a 24-well plate until they reached full confluency, and incubated with serum-free medium overnight. Staining for C3b was performed as described in [4], with a few changes. Cells were incubated for 30 min at 37°C with 10% C9-depleted NHS and 150 µg/ml of CRP, supplemented with a physiological concentration of a particular C2 variant. Detection of C3b and CD31 was conducted with anti-C3c-FITC (Dako)/CD31-PE antibodies (Sigma-Aldrich) diluted 1:200 in 0.1% BSA in PBS for 30 min in 4°C. Cells were washed with PBS and gently detached with enzyme-free cell dissociation solution (Merck Millipore) to be analyzed by flow cytometry using CytoFLEX (Beckman Coulter) or left on the plate and overlaid with a mounting medium with TRITC-Phalloidin (Vectashield) for fluorescent microscopy imaging (Olympus IX83).

### C3b Deposition Measurement by ELISA

ELISA plates (Nunc MaxiSorp ™, ThermoFisher) were coated with human CRP (Sigma) or a preparation of human immunoglobulins (Pentaglobin^®^, Biotest) overnight at 4°C and then blocked for 1 h with 3% BSA (Sigma). Afterward, the plates were incubated with 0.25% NHS, and the particular C2 variant was diluted in GVB++ buffer (5 mM veronal buffer, 0.1% gelatin, 145 mM NaCl, 1 mM MgCl_2_, 0.15 mM CaCl_2_) and incubated for 30 min at 37°C with mild shaking. C3b protein (Complement Technology) serially diluted in NHS and coated on the plate instead of CRP was used for the preparation of the standard curve. C3b detection was performed with polyclonal goat anti-human C3 antibody (Complement Technology), followed by rabbit anti-goat antibody conjugated with HRP (Dako, Glostrup, Denmark) diluted 1:10,000 and 1:5,000 in PBS, respectively. The assay was developed by using 3,3′,5,5′-Tetramethylbenzidine (Sigma Aldrich) according to the manufacturer’s instructions.

### Molecular Modeling and Bioinformatics Analysis

A three-dimensional model of C2 protein was built with SWISS-MODEL programme (9), using the structure of Factor B protein as a template (PDB code 2XWB) (10). For assessing amino frequency occurrence in position 249, we used the same methods and data set as it is described in reference (2). Amino acid sequences of C2 and FB proteins were retrieved from the UniProt amino acid sequence database searched for the term *complement C2*, and the results were further manually filtered out to obtain only sequences of C2 and FB proteins. For further analysis, 151 sequences were selected. All selected sequences were subjected to multiple sequence alignment using a fast Fourier transform algorithm.

## Results

Clinical synopsis of patients with R249C and S250C mutations and additional genetic data are described in the supplementary material section and in **Table S1**.

**Figure 1:**
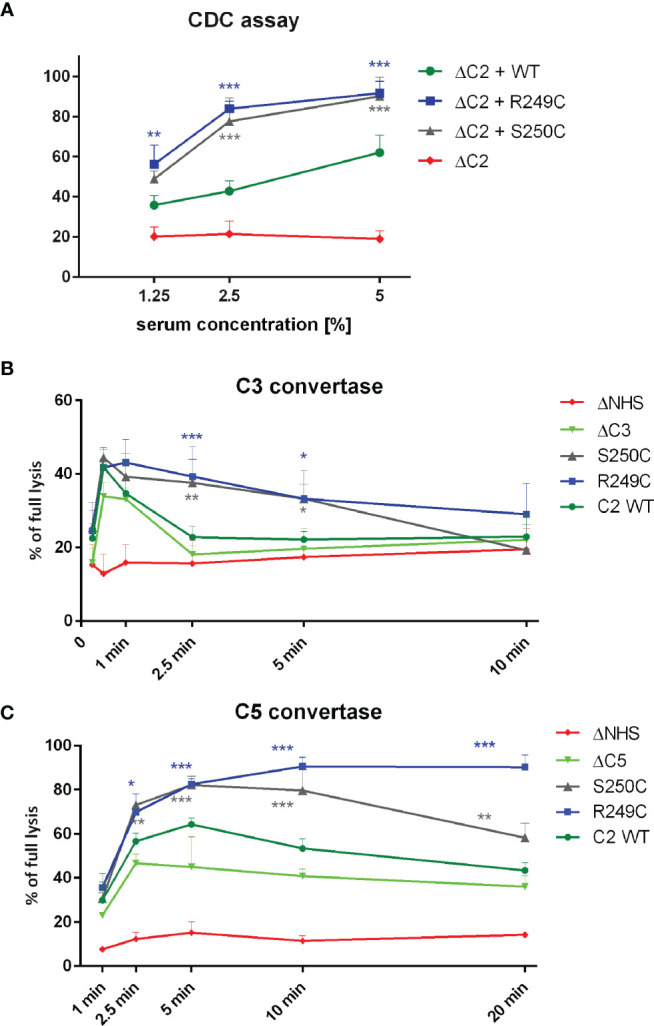
Functional assays of C2 variants. **(A)** CDC assay. Calcein-loaded Raji cells sensitized with anti-CD20 mAb (ofatumumab) were suspended in C2-depleted serum (ΔC2) supplemented with physiological concentration of C2 protein. Cells were incubated for 30 min at 37°C. Supernatant was collected, and fluorescence of released calcein was measured. The readout obtained for cells lysed with 30% DMSO was considered as full lysis, and the readout obtained for cells incubated with ΔC2 serum alone indicated background lysis (negative control). **(B, C)** Convertase activity assays for classical C3 convertase **(B)** and classical C5 convertase **(C)** were performed on calcein-loaded Ramos cells suspended in 15% of C3-depleted serum (ΔC3) **(B)** or C5-depleted serum (ΔC5) **(C)** supplemented with physiological concentration of C2 variants. After indicated time period, cells were washed with EDTA-containing buffer to disable further convertase formation and then suspended in 5% guinea pig serum diluted in the same EDTA buffer. The readout was performed as in CDC assay, but heat-inactivated serum (ΔNHS) instead of ΔC2 served as a negative control. Data are collected from at least three experiments. Symbols *, **, and *** denote statistically significant differences *vs.* WT supplementation, at p levels of 0.05, 0.01, and 0.001 according to Dunn’s multiple comparison test.

Recombinant, His-tagged wild-type (WT), S250C, and R249C C2 variants were purified as shown in **Figure S1**. Functional assays testing the activity of C2 proteins revealed that supplementation of C2-depleted serum (ΔC2) with S250C or R249C variants exerted significantly higher CDC than supplementation with WT C2 (**Figure 1A**). CP/LP C3 convertase formed in the presence of each mutated C2 variant showed prolonged activity compared to the enzyme formed upon serum supplementation with WT protein (**Figure 1B**), whereas C5 convertase showed either higher amplitude or prolonged activity (**Figure 1C**). Notably, the GoF character of the R249C mutation was more pronounced than that of S250C. The differences in CDC between the R249C and S250C mutants and the WT protein were eliminated by using Raji cells devoid of CD55 complement inhibitor as a target (**Figure S2**), which illustrates that convertase stability is an important factor for the observed phenotype. Afterward, serum samples collected from both patients carrying GoF mutations were tested in the CDC assays similar to these using ΔC2 serum supplemented with recombinant C2 proteins. Patients’ sera performed weaker than NHS (**Figure S3A**), but when mixed 1:1 with ΔC2 NHS, their cytotoxic activity reached the levels comparable (patient with S250C mutation) or significantly higher (patient with R249C mutation) than NHS mixed with the same ΔC2 serum (**Figure S3B**). On one hand, the observed differences in the cytotoxic activity of unmixed sera show the overall complement activity of patients’ sera lower than normal. On the other hand, the performance of patients’ sera after mixing with ΔC2 NHS suggests the presence of the C2 variant capable of enhancing the CP activity once the other, putatively exhausted elements of the complement cascade, are restored.

**Figure 2:**
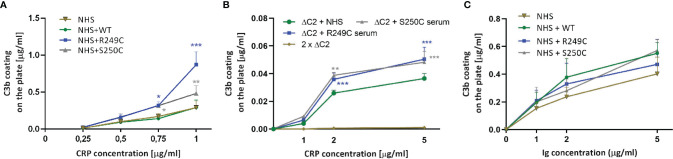
C3b deposition assay in the presence of CRP and human Ig preparation. **(A)** Increasing concentrations of C-reactive protein (CRP) were coated onto the ELISA microplate and overlaid with 0.25% normal human serum (NHS) +/− recombinant C2 variants. After 30 min incubation, C3b deposition was detected by anti-C3b antibody. Purified C3b directly coated on the plate was used as a standard. **(B)** The same assay was performed with a 1:1 mix of ΔC2 serum with patients’ sera or NHS as a control. **(C)** ELISA plates were coated with a preparation of human Ig (pentaglobin) instead of CRP and overlaid with NHS with recombinant C2 variants. Differences between the given C2 variant and the WT were analyzed by Dunnett multiple comparison test for non-repeated measures. *P < 0.05; **P < 0.01; ***P < 0.001.

Retrospective analysis revealed the presence of an increased level of CRP in serum collected from a patient with S250C mutation (see **Supplementary Data**). Therefore, we analyzed whether the CP initiated by CRP can be enhanced by the S250C and R249C variants of C2. ELISA plates were coated with increasing concentrations of CRP and overlaid with NHS +/− recombinant C2 WT or R249C or S250C variants. A threshold of CRP coating was observed, beyond which the rate of C3 deposition in GoF C2 variant-containing sera increased significantly higher than in non-supplemented serum or serum supplemented with WT C2 (**Figure 2A**). The same effect was observed when a 1:1 mix of patient sera (or NHS as a control) with ΔC2 serum was used (**Figure 2B**). Notably, there was no significant enhancement of C3 deposition by recombinant C2 variants compared to the WT protein when CP was initiated by the preparation of human immunoglobulins (**Figure 2C**).

**Figure 3:**
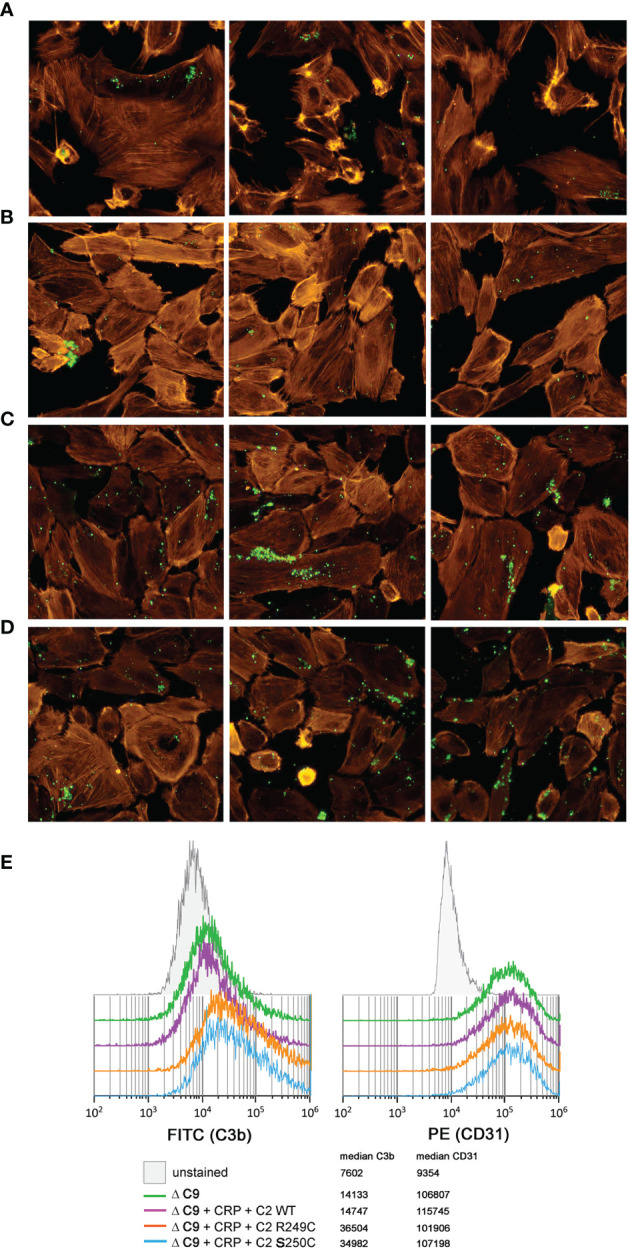
C3b deposition on glomerular endothelial cells. Immortalized glomerular endothelial cells were seeded onto glass chambers and exposed to C9-depleted human serum (ΔC9) in order to prevent lysis. Deposition of C3b (green) was analyzed by fluorescent microscopy **(A–D)** when nothing **(A)**, CRP + WT C2 **(B)**, CRP + R249C variant **(C)**, or CRP + S250C variant **(D)** was added. Labeled phalloidin (orange) was used as a counterstaining. Three independent images are shown for each condition. **(E)** To perform an alternative, quantitative analysis that confirms the observed pattern of immunofluorescence, cells were harvested and examined by flow cytometry for both C3b deposition (left histogram) and CD31 expression (right histogram). Results show 10,000 events, previously gated to eliminate cell doublets and cell debris.

The addition of either S250C or R249C C2 variants, but not of WT C2, to C9-depleted NHS supplemented with a high concentration of C-reactive protein (CRP) significantly increased the deposition of C3 on the surface of glomerular endothelial cells (**Figure 3**). However, a similar effect was reproduced when C9-depleted NHS was supplemented only with recombinant C2 variants but no CRP (**Figure S4**) but could not be observed when a 1:1 mix of ΔC2 serum and patients’ sera were used (not shown).

**Figure 4:**
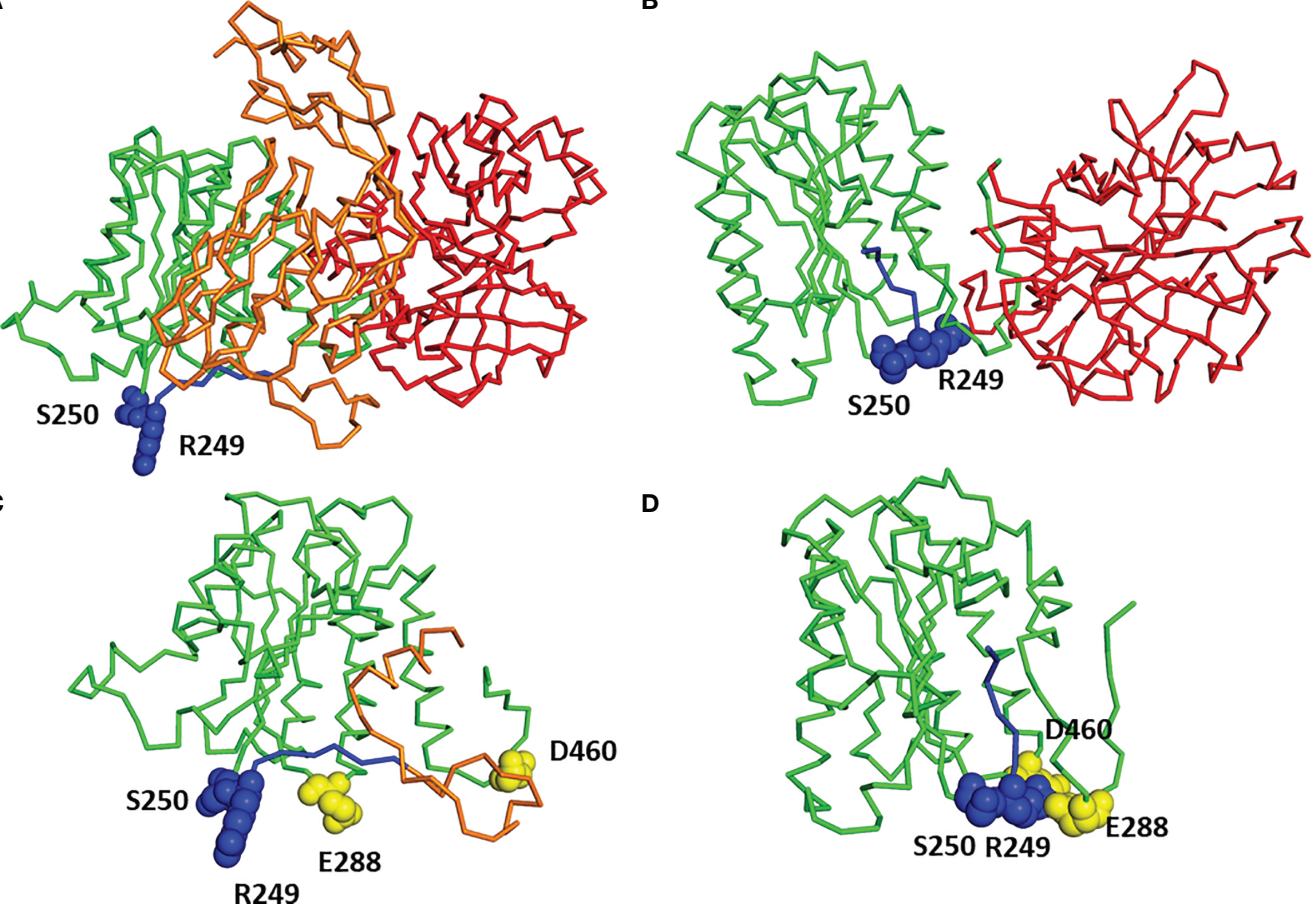
Molecular modeling of C2 and C2b. **(A)** Three-dimensional model of full C2 sequence built by homology modeling using Factor B structure (PDB code 2XWB) as a template. C2b domain in orange, VWA domain in green, SP domain in red, residues 243–250 in blue, and residues 249 and 250 in spheres representation. **(B)** Three-dimensional structure of C2a (PDB code 2I6Q). VWA domain in green, SP domain in red, residues 243–250 in blue, and residues 249 and 250 in spheres representation. **(C)** Close-up view of VWA domain and part of domain C2b from the complete structure of C2 protein [see **(A)**], color code as used in **(A)**, moreover residues 288 and 460 are shown in yellow. **(D)** Close-up view of VWA domain from C2a structure [see **(B)**] with residues 288 and 460 shown in yellow.

Molecular modeling, based on the available structural data, indicates that the C2a domain undergoes spatial reorganization upon dissociation of the C2b domain (**Figures 4A, B**), as previously suggested by others (11). The importance of the region including the mutated residues for the function of C2 protein is emphasized by the strong conservation of residues occurring at positions 250 (2) and 249 (see **Table S2**). In the unprocessed C2 protein, the 243–250 region is unstructured and exposed to solvent, possibly to facilitate access of the C1s protease. Upon cleavage, this fragment is relocated inside the von Willebrand factor A (VWA) domain of C2a, taking the place previously occupied by the C-terminal helix of the C2b domain (**Figure 4**). In addition, the VWA domain acquires a more compact conformation in the C2a structure (**Figure 4D**) than when it is a part of the full C2 (**Figure 4C**). Residues 250 and 249 are located in the “hinge” region that may regulate the relocation of the 243–250 fragment (see **Figures 4C, D**, fragment marked in blue) following the cleavage of the C2 protein. The serine 250 is highly conserved in the amino acid sequences of both C2 and factor B (2). For residue 249, the evolutionary conservation depends on the type of protein; for the C2 sequences it is the arginine; and for the factor B sequences, it is the proline (see **Table S2**). Analysis of the structure of C2a showed that the arginine 249 is in the immediate spatial vicinity of the residues E288 and D460 (**Figure 3D**), which are also evolutionary conserved within C2 sequences. Conformational changes in the VWA domain upon the cleavage of C2 and release of serine protease activity could be related to the interaction between the triad of amino acid residues from positions 249, 288, and 460. Mutations reported in the current study, especially R249C, may interfere with the electrostatic character of such interaction and intramolecular dynamics associated with the C2-C2a rearrangement.

## Discussion

In contrast to mutations in AP convertase components, the causative role of mutations in their CP/LP counterparts in the course of complement-mediated renal diseases is not straightforward. Similar to the case of the aHUS patient carrying the S250C mutation in C2 (2), there are reports of C4NeF autoantibodies stabilizing CP/LP convertases as a sole, potentially pathogenic factor identified in patients with C3 glomerulopathies (12, 13). However, the information about specific triggers that could initiate CP or LP [e.g., other autoantibodies, elevated level of pentraxins (14, 15)] was not available in the abovementioned cases and is still an intriguing question. In the current study, we report a new C2 GoF mutation R249C in a C3G without anti-FH, C3NeF, and most common autoantibodies. This finding imposed the question of whether non-Ig stimuli can act as a driver of the excessive glomerular C3 deposition (including the subendothelial space), which is characteristic in C3G-affected individuals (16), as well as for the C3 deposition in the renal microvascular endothelium cell lining in aHUS patients (17, 18). We chose to study the impact of CRP, as this acute phase protein may directly interact with endothelial cells (19, 20) and has the ability of direct CP activation by binding C1q (4). The observation that aHUS is often preceded by bacterial infections (21) and that a substantial percentage of C3G patients presents with high titers of antistreptolysin-O (22) make CRP a strong candidate for being a trigger of complement dysregulation in carriers of C2 GoF mutations. Indeed, our data reveal the possibility that combined action of CRP and GoF C2 mutations results in elevated complement deposition in kidney microvasculature and as such grounds a pathogenic process. Our *in vitro* experiment demonstrated that the amounts of C3 deposited upon CP stimulation by CRP are significantly higher when the convertases are formed by GoF C2 variants.

One of the limitations of our study is the inability to judge what was the initial trigger of complement activation in patients. Serum collected at the time of genetic analyses (which was not necessarily the exact time of admission) from the carrier of S250C mutation contained an elevated concentration of CRP (22.6 μg/ml), whereas serum from the other patient contained CRP within the normal range (2.2 μg/ml). Both patients were negative for antistreptolysin-O, an indicator of common infections in the nearby past. Yet, it may be impossible to retrospectively assess the CRP status at admission, as it peaks within 2 days, and its half-life is 18–20 h (23). Moreover, even though both patients were registered in the Spanish aHUS/C3G Registry, the C3G diagnosis of the patient carrying S250C mutation is uncertain. Besides the intense staining for C3 deposits, the biopsy also revealed non-specific chronic changes and immune deposits, including IgM antibodies. Therefore, the C3 deposition detected in the biopsy might be due to complement activation on immunoglobulin complexes. More to this end, this patient was retrospectively diagnosed with ANA autoantibodies, which could theoretically drive the CP activation. We observed an elevated C3 deposition on glomerular endothelial cells incubated with C9-depleted NHS containing recombinant GoF C2 variants but no additional CRP (**Figure S4**). It is possible that NHS, which was prepared by pooling of serum collected from many different individuals, contains natural IgM antibodies or complement-activating antibodies bondable to polymorphic determinants present on the immortalized glomerular endothelial cells. To sum up, we do not exclude the antibody-mediated complement activation leading to C3 deposition in glomeruli of both patients. We show that a possibility of CP initiation by a high concentration of CRP, which results in an increased C3 deposition, exists in carriers of GoF C2 mutations. Importantly, a certain threshold of CRP must be reached to significantly elevate deposition of C3, as evidenced by ELISA assays (**Figures 2A, B**) and the fact that we could not see this effect on glomerular endothelial cells when patient serum containing 22.6 μg/ml was used. However, the CRP concentration of 150 μg/ml used in the immunofluorescence imaging and flow cytometry experiments is not unusual and can be achieved in case of infections but also non-infectious inflammatory lesions, as reported in (24–27). Another lesson from our experiments is that the GoF phenotype of both C2 variants can be observed when CRP but not human immunoglobulins are coated on ELISA plates (**Figure 2**). This observation is in line with our previous study suggesting a critical role of human membrane-bound inhibitors, as no effect of S250C mutation was observed either on aggregated immunoglobulins coated on ELISA plate or the surface of sensitized sheep erythrocytes (2). A possible explanation may involve a biphasic action of CRP in CP activation: the first phase is the recruitment of C1 complex, and the second phase is binding of soluble complement inhibitors, which provide protection beyond the level attainable for the normal activity of the fluid-phase inhibitors in serum (28). The ability to override such protection is one more argument to study CRP in the context of GoF C2 variants.

Here we provided evidence that two C2 mutations, which are located in the short motif located between C2a/C2b cleavage site (residue 243) and VWA domain (residues 254–452), result in the formation of the more stable and processive CP/LP convertases. Based on the large numbers of identified GoF variants in AP convertase components (factor B, C3) associated with aHUS and C3G (3, 7, 18, 29) and the substantial similarity between AP and CP/LP convertases components (C2, C4), this should not be surprising, but has only recently been suggested (2). However, the placement of the identified mutations within the C2 sequence is not obvious, as could be anticipated from known GoF variants of homologous factor B. Mutation of serine to cysteine is the most conservative change in amino acids, as these two residues differ only in γ-atom (sulfur instead of oxygen); still, it can impact the propensity of hydrogen bond formation (30). Although the substitution of arginine for cysteine at position 249 introduces a difference in potential electrostatic/polar interactions, this region does not appear to be directly involved in interactions with complement inhibitors of RCA (regulator of complement activity) family (31, 32). We postulate that substitutions at positions 249 and 250 may impact the hinge region of the N-terminal VWA domain, which is engaged in intramolecular interactions with other patches that determine the more compact structure of the C2a fragment compared to unprocessed C2 molecule. We identified a triad of evolutionarily conserved amino acids—R249, E288, and D460—localized in immediate spatial vicinity. Notably, in the factor B sequences, the positions corresponding to the 288 and 460 in the human C2 protein are strongly conserved too but occupied by different amino acids: glycine (position 288) and leucine (position 460), whereas the residue 249 is proline (**Table S2**). Therefore, the interaction itself, not its character, seems to be evolutionarily preserved: in the case of the C2 sequence it is an electrostatic effect (arginine, aspartic acid, glutamic acid), while in the case of factor B sequences it is a hydrophobic effect (proline, glycine, leucine). The hypothesis related to the evolutionarily conserved interactions described above, as well as the role of these interactions in the functioning of C2a, should be verified by appropriate biochemical studies.

An important conclusion of our studies is that routine genetic diagnostic in patients with a clinical diagnosis of aHUS and C3G must include the genes encoding the CP/LP components. Assays detecting C4NeF, which are not as common as C3NeF assays, should also be included in the analysis of C3G patients (33). However, we also report that acute phase proteins that initiate CP in an Ig-independent way may trigger complement dysregulation in carriers of the GoF C2 variants. Confirmation of the aforementioned scenario and identification of more mutational hotspots in C2 may bring new directions in the management of such C3G and aHUS cases, e.g., eradication of microbial pathogens or autoreactive B cell clones, as well as close monitoring for inflammatory mediators or increased titers of autoantibodies. In the context of a precision medicine, the identification of renal patients with underlying CP/LP dysregulation would suggest that these cases might be better treated with CP inhibitors, rather than C3 or C5 inhibitors.

## Data Availability Statement

The raw data supporting the conclusions of this article will be made available by the authors, without undue reservation.

## Ethics Statement

Ethical review and approval was not required for the study on human participants in accordance with the local legislation and institutional requirements. The patients/participants provided their written informed consent to participate in this study.

## Author Contributions

Conceptual work: SRC and MO. Experimental work and data acquisition: AU, DK, AK, GS, AS, EA, MT, and SO. Preparation of crucial molecular tools/models: IJ, RS, SS. Patients’ diagnosis: ECA, MS. Writing and critical review of the manuscript: AU, DK, GS, IJ, RS, SS, SRC, and MO. All authors contributed to the article and approved the submitted version.

## Funding

The project was funded by National Science Centre Poland grant nos. 2015/18/M/NZ6/00334 and 2018/29/N/NZ6/01413. SRC was supported by grants from the Spanish Ministerio de Economía y Competitividad-FEDER (PID2019-104912RB-I00) and Autonomous Region of Madrid (S2017/BMD-3673). Computational resources used in this project were provided by the Informatics Center of the Metropolitan Academic Network (IC MAN-TASK) in Gdańsk.

## Conflict of Interest

SRC performed genetic analyses of aHUS patients for Alexion in the framework of contract and obtained honoraria for lectures/presentations.

The remaining authors declare that the research was conducted in the absence of any commercial or financial relationships that could be construed as a potential conflict of interest.

## Publisher’s Note

All claims expressed in this article are solely those of the authors and do not necessarily represent those of their affiliated organizations, or those of the publisher, the editors and the reviewers. Any product that may be evaluated in this article, or claim that may be made by its manufacturer, is not guaranteed or endorsed by the publisher.
