# Supplementary material for: Gain-of-Function Mutations R249C and S250C in Complement C2 Protein Increase C3 Deposition in the Presence of C-Reactive Protein
Source: Front Immunol. 2021 Nov 25;12:724361. doi: 10.3389/fimmu.2021.724361 (PMC8654806; doi:10.3389/fimmu.2021.724361)
Supplement: Supplementary file 1 [file DataSheet_1.pdf]

SUPPLEMENTARY RESULTS

Table S1 Characteristics of patients with identified GoF mutations in C2

Position on chromosome 6	ID	cDNA substitution	Amino acid substitution	Diagnosis of patients with mutation	Genotype	MCP risk polymorphism	CFH risk polymorphism	Other potential pathogenic complement genetic variants	Anti FH antibodies	C3Nef	Autoantibodies
31901972	rs771081314	c.C745T	p.R249C	C3GN	HET	NO	HET	p.S115F:C1S:HET; p.D315N:C1S:HET	No	No	No
31901976	rs150827255	c.C749G	p.S250C	C3GN ??	HET	HET	NO	None	No	-	ANA #
31901976	rs150827255	c. C749G	p.S250C	aHUS *	HET	HOM	NO	None	-	-	-

HET – heterozygous, HOM – homozygous, ANA – anti-nuclear antibodies, * - patient reported by *Urban et al.*, # - ANA were confirmed 6 years after the initial admission

Table S2. Occurrence (in percent) of the most frequent amino acid types in selected positions

Amino acid position in C2 protein (Homo Sapiens)	Amino acid type occurrence	Remarks
250	S>99%	Data from reference 3
249	R 60%, K 3%	Observed mostly for C2 amino acid sequences
	P 30%	Observed mostly for Factor B amino acid sequence.

Fig. S1 Purification of C2 variants

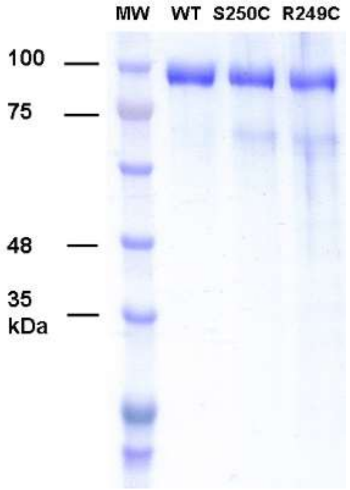

Purified C2 variants (2 μg) were run on SDS-PAGE under reducing conditions and stained with Coomassie blue. Faint band below 70 kDa corresponds to C2a fragment, which occurred during spontaneous degradation, as concluded from Western Blotting (not shown).

Fig. S2 CDC assay on CD55 wild-type (WT) and CD55 KO Raji cells.

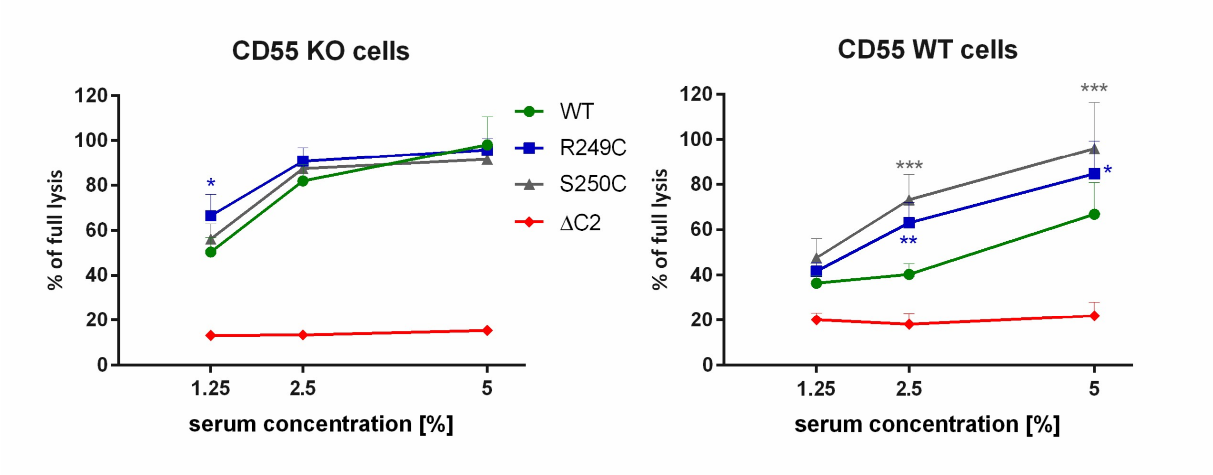

Raji CD55-knockout cells and control Raji cells were used for complement-dependent cytotoxicity (CDC) assay in ΔC2 serum supplemented with selected C2 mutants. All graphs show the results obtained from three independent experiments. Statistical significance of differences between the given C2 variant and the WT was analyzed by using the Dunnett multiple comparison test for non-repeated measures. *P < 0.05; ***P* < 0.01; ****P* < 0.001.

Fig.S3 CDC assay on Raji cells incubated with patient sera.

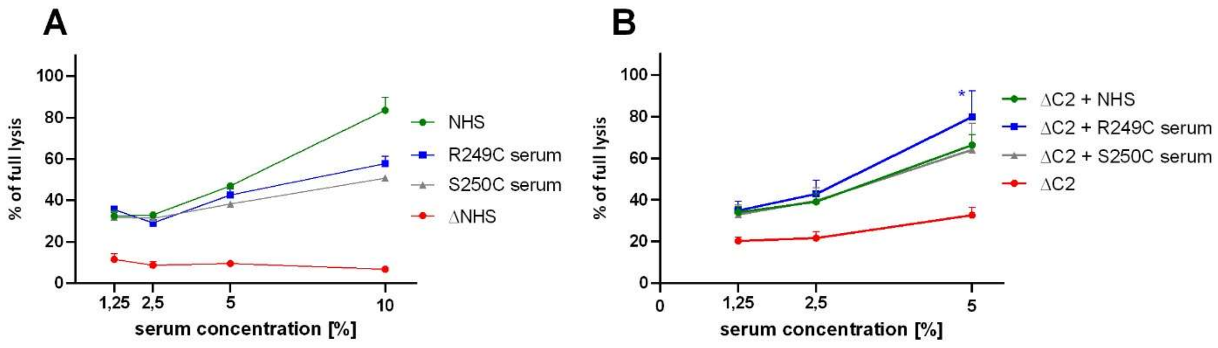

The CDC assay was performed as described in Fig. 1 but unmixed patient sera (or NHS) (panel A) or patient sera mixed 1:1 with Δ C2 serum (panel B) were used instead of ΔC2 serum supplemented with recombinant C2 proteins. Panel A shows the results of single experiment performed in duplicates whereas panel B shows the results from three independent experiment. Statistical significance of differences between the given C2 variant and the WT was analyzed by using the Dunnett multiple comparison test for non-repeated measures. *P < 0.05

Fig. S4 Flow cytometry analysis of C3 deposition in glomerular endothelial cells.

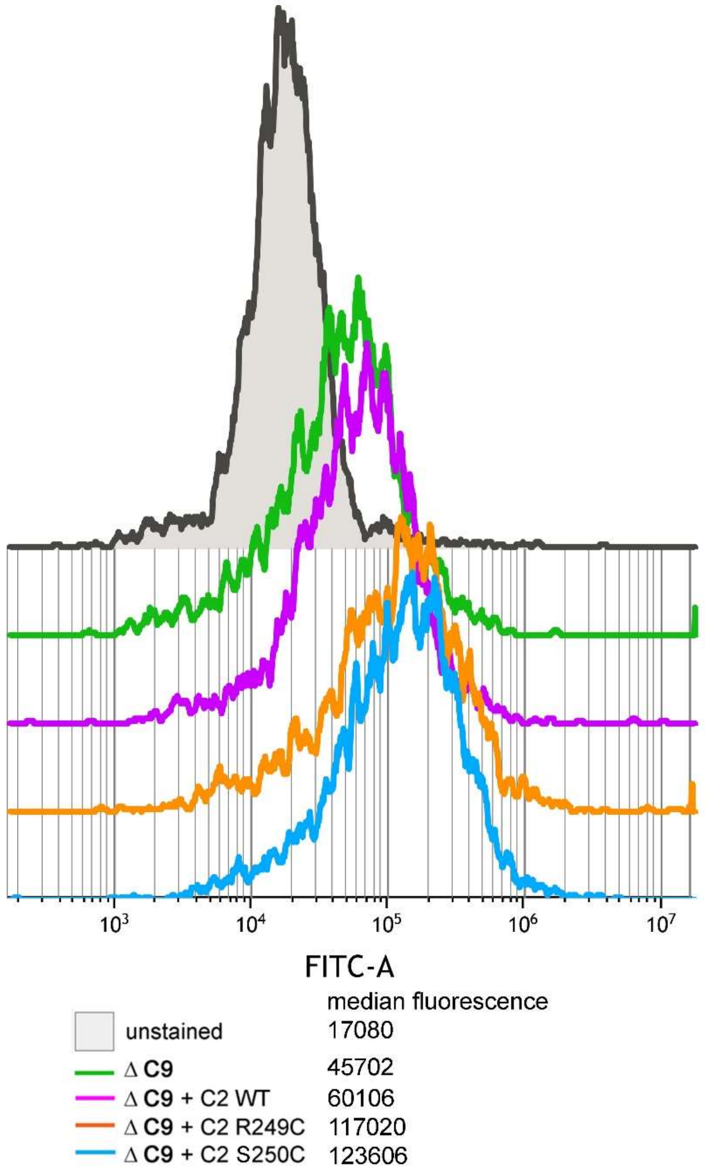

Glomerular endothelial cells were harvested and examined by flow cytometry for C3 deposition, as in Fig. 3, but no CRP was added to NHS supplemented with WT or GoF variants of C2 protein. The results show ten thousand events, previously gated to eliminate cell doublets and cell debris. Histograms present the results of one out of two experiments performed, in which principally the same results were obtained.

Clinical synopsis of the C3G patient carrying the C2 p.S250C variant

He is a 51y-old male, with a diagnosis of Type 2 diabetes, who in 2010 was admitted to the clinic with a proteinuria in the nephrotic range that evolved to chronic renal insufficiency. He had no familial history of renal disease. Studies performed in 2016 showed a normal complement profile with a positive for anti-nuclear antibodies, but no anti DNA antibodies were detected. Monoclonal gammopathy was excluded as no paraprotein/monoclonal proteins were detected.

A biopsy was performed with about 15 glomeruli useful for diagnosis, 8 of them were sclerosed in wafer-like shape. In the rest, an increase in global and diffuse mesangial matrix stand out, with mild mesangial hypercellularity and presence of segmental sclerosis with capsular adhesion affecting the vascular pole in 3 glomeruli. Occasional double contours were observed. No endocapillary or extracapillary proliferation was observed. Staining for Congo Red was negative. In the interstitium there was an extensive fibrosis affecting 40% of the cylinder with accompanying tubular atrophy. Three vascular sections with moderate intimal fibrosis were identified. Cryostat sections incubated with anti-IgG, anti-IgA, anti-IgM, anti-C1q, anti-C3, kappa and lambda sera labeled with fluorescence. Global and diffuse, linear deposits were observed in handles and mesangium with zones of subendothelial pattern for C3 (++) and kappa (++) and lower intensity for lambda (+) and IgM (+). Immunofluorescence was negative with the other antisera. Overall, it was a biopsy in which different lesions were observed. There was a clear predominance of nonspecific chronic changes, with 53% glomerular sclerosis and 40% of interstitial and tubular fibrosis and an important arteriosclerotic atrophy. Additionally, lesions of diffuse intercapillary glomerulosclerosis, of nephroangiosclerosis, focal and segmental glomerulosclerosis, as well as immune deposits compatible with C3 nephropathy were observed. The patient experienced a progressive loss of renal function that took him to hemodialysis on Nov, 2018. He was then referred to a main hospital to be included in the waiting list for kidney transplantation. In Feb 2020 he received a cadaver kidney transplant and up today presents normal kidney function.

Genetic studies of the complete complement gene set were performed in 2019 and the C2 p.S250C variant was the only relevant alteration found in the patient. He carries the *MCPggaac* polymorphism in heterozygosis and is negative for the *CFH-H3* aHUS risk polymorphism. At the time of these analyses, levels of C3, C4 and FH were within the normal range.

Clinical synopsis of the C3G patient carrying the C2 p.R249C variant

He is a 59y-old male with a previous history of nonspecific sediment alterations. He presented with a creatinine of 1.3mg/dl and FGxMDRD-4 57ml/min, microscopic hematuria and a proteinuria in the nephrotic range that flared up to chronic kidney disease. He was negative in the autoimmunity study and for the HBV, HCV and HIV serology.

A biopsy was performed that included seven glomeruli with sclerosis in three of them. The remaining showed mesangial hypercellularity with focal endocapillary proliferation. No intraglomerular inflammatory cells were observed. There were synechiae to the Bowman capsule and thickening of the glomerular basement membrane at some points. The interstitium presented a chronic inflammatory infiltrate that surrounded the sclerosed glomeruli and the tubules. Atrophy was also observed in part of the tubules and there were also accumulations of intraluminal polynuclear cells. The infiltrate also presented some plasma cells. Congo red staining was negative. The vessels showed moderate arteriosclerotic changes. In the immunofluorescence there was mesangial deposition of C3 (++) in the glomeruli without IgA, IgG, IgM or C1Q deposits. There was also absence

of C4d deposits in the glomeruli, but few C5b9 deposits. Another biopsy on September 2020 showed mesangial and intramembranous dense deposits in the electron microscopy. Currently he is under treatment with MMF and corticoids, and presents normal levels of C3. Monoclonal gammopathy was excluded as no paraprotein/monoclonal proteins were detected.

Genetic studies of the complete complement gene set were performed in 2018 revealing the C2 p.R249C variant and two additional changes in the *C1S* gene (p.S115F, rs138764697; p.D315N; rs117907409), which significance is uncertain. He carries the *CFH-H3* polymorphism in heterozygosis and is negative for the *MCPggaac* aHUS risk polymorphism. At the time of the genetic analysis, levels of C3, C4 and FH were within the normal range.

Notably, this patient has a brother presenting proteinuria and microhematuria. Glomerular filtration rate was slightly affected (65ml/min/1.73 m^2) and creatinine was 1.3mg/dl. The brother carries the same pattern of heterozygous mutations in *C2* and *C1S* genes. A kidney biopsy is considered but has not been scheduled yet.
